# Is self-guided internet-based cognitive behavioural therapy (iCBT) harmful? An individual participant data meta-analysis

**DOI:** 10.1017/S0033291718000648

**Published:** 2018-03-15

**Authors:** Eirini Karyotaki, Lise Kemmeren, Heleen Riper, Jos Twisk, Adriaan Hoogendoorn, Annet Kleiboer, Adriana Mira, Andrew Mackinnon, Björn Meyer, Cristina Botella, Elizabeth Littlewood, Gerhard Andersson, Helen Christensen, Jan P. Klein, Johanna Schröder, Juana Bretón-López, Justine Scheider, Kathy Griffiths, Louise Farrer, Marcus J. H. Huibers, Rachel Phillips, Simon Gilbody, Steffen Moritz, Thomas Berger, Victor Pop, Viola Spek, Pim Cuijpers

**Affiliations:** 1 Department of Clinical Psychology, VU Amsterdam and Institute for Public Health Research, Amsterdam, the Netherlands; 2 Department of Psychiatry, GGZ inGeest and VU University Medical Centre, Amsterdam Public Health research institute, Amsterdam, the Netherlands; 3 Department of Epidemiology and Biostatistics and Amsterdam Institute for Public Health Research, VU University Amsterdam, Amsterdam, the Netherlands; 4 Department of Psychology and Technology, Jaume University, Castellon, Spain; 5 CIBER Fisiopatología Obesidad y Nutrición (CIBERobn), Instituto Salud Carlos III, Spain; 6 Black Dog Institute and University of New South Wales, Prince of Wales Hospital, Sydney, Australia; 7 Center for Mental Health, University of Melbourne, Melbourne, Australia; 8 Research Department, Germany and Department of Psychology, City University, Gaia AG, Hamburg, London, UK; 9 Department of Health Sciences, University of York, York, UK; 10 Department of Behavioural Sciences and Learning, Sweden Institute for Disability Research, Linköping University, Linköping, Sweden; 11 Department of Clinical Neuroscience, Psychiatry Section, Karolinska Institute for Disability Research, Stockholm, Sweden; 12 Department of Psychiatry and Psychotherapy, Lübeck University, Lübeck, Germany; 13 Department of Psychiatry and Psychotherapy, University Medical Center Hamburg-Eppendorf, Hamburg, Germany; 14 Institute of Mental Health, University of Nottingham, Nottingham, UK; 15 Research School of Psychology, College of Biology, Medicine & Environment, Australian National University, Canberra, Australia; 16 Centre for Mental Health Research, The Australian National University, Canberra, Australia; 17 Department of Primary Care and Public Health Sciences, King's College London, London, UK; 18 Department of Clinical Psychology and Psychotherapy, University of Bern, Bern, Switzerland; 19 CoRPS – Center of Research on Psychology in Somatic diseases, Tilburg University, Tilburg, the Netherlands

**Keywords:** Depression, iCBT, internet-based treatment, self-guided psychotherapy

## Abstract

**Background:**

Little is known about potential harmful effects as a consequence of self-guided internet-based cognitive behaviour therapy (iCBT), such as symptom deterioration rates. Thus, safety concerns remain and hamper the implementation of self-guided iCBT into clinical practice. We aimed to conduct an individual participant data (IPD) meta-analysis to determine the prevalence of clinically significant deterioration (symptom worsening) in adults with depressive symptoms who received self-guided iCBT compared with control conditions. Several socio-demographic, clinical and study-level variables were tested as potential moderators of deterioration.

**Methods:**

Randomised controlled trials that reported results of self-guided iCBT compared with control conditions in adults with symptoms of depression were selected. Mixed effects models with participants nested within studies were used to examine possible clinically significant deterioration rates.

**Results:**

Thirteen out of 16 eligible trials were included in the present IPD meta-analysis. Of the 3805 participants analysed, 7.2% showed clinically significant deterioration (5.8% and 9.1% of participants in the intervention and control groups, respectively). Participants in self-guided iCBT were less likely to deteriorate (OR 0.62, *p* < 0.001) compared with control conditions. None of the examined participant- and study-level moderators were significantly associated with deterioration rates.

**Conclusions:**

Self-guided iCBT has a lower rate of negative outcomes on symptoms than control conditions and could be a first step treatment approach for adult depression as well as an alternative to watchful waiting in general practice.

## Introduction

Depression is a common and major health issue that is associated with a considerable personal and societal burden (Reddy, 2010; Lepine & Briley, 2011). Self-guided forms of internet-based cognitive behaviour therapy (iCBT) can increase accessibility and reduce the costs of depression treatment (Hedman *et al.* 2012; Muñoz *et al.* 2015). Over the past decade, free or commercially provided self-guided iCBT programmes have been made available to individuals with depression (Kaltenthaler *et al.* 2006). However, there is an ongoing discussion about the advantages and disadvantages of such programmes (Andersson & Titov, 2014). Many healthcare systems remain hesitant to implement self-guided iCBT. Among the barriers to implementation are concerns regarding safety and effectiveness (Waller & Gilbody, 2009). Offered as first stage treatment, self-guided iCBT has been criticised as potentially delaying individuals suffering from depression receiving effective clinical services (Robinson *et al.* 1998; Ybarra & Eaton, 2005).

Our recent individual participant data (IPD) meta-analysis demonstrated that self-guided iCBT produces encouraging effects although average effects are small in magnitude (Karyotaki *et al.* 2017). Small effects can be clinically useful and relevant, for instance in countries where mental healthcare services are scarce or inaccessible for other reasons (Muñoz *et al.* 2015; Karyotaki *et al.* 2017). Despite the ample evidence regarding the overall benefits of iCBT, possible effects harmful to individuals have been infrequently monitored. As with any other potentially effective treatment, iCBT could also result in undesirable outcomes or fail to arrest substantial ongoing deterioration (Dimidjian & Hollon, 2010). The issue of negative effects is crucial for clinical decision-making, yet limited systematic examination of this issue exists for iCBT.

In contrast to the vast majority of pharmacological trials, which rigorously measure and report adverse outcomes, only half of psychotherapeutic trials report undesirable outcomes, in particular substantial symptom deterioration (Jonsson *et al.* 2014; Vaughan *et al.* 2014). Previous research has shown that some forms of psychotherapy can be hazardous for some patients e.g. prolonged imaginal exposure may result in worsening of symptoms in some patients with posttraumatic stress disorder (Foa *et al.* 2005). With regard to self-guided psychological treatment, it has been argued that it may not be appropriate for all individuals (Newman, 2000). For instance, self-guided interventions may not be intensive enough for individuals with severe symptoms (Mohr *et al.* 1990). Moreover, lack of therapeutic support may jeopardise therapy outcomes as the progress of patients is not monitored (Newman *et al.* 2003). Most self-guided interventions are not tailored to the current depressive status of the individual and, accordingly, do not respond to symptom deterioration (Andersson & Titov, 2014).

To our knowledge symptom deterioration has not been examined in self-guided iCBT to date. It is therefore clearly important to determine the prevalence and extent of symptom deterioration in self-guided iCBT. Furthermore, given that not everyone receiving self-guided iCBT experiences worsening of symptoms, research examining for whom this particular form of therapy may be harmful is sorely needed: individuals likely to deteriorate could be diverted to other more appropriate treatment options. However, study-level meta-analyses and randomised controlled trials (RCTs) cannot thoroughly examine moderators of deterioration due to inadequate power (Bower *et al.* 2013). Novel methodological approaches, such as IPD meta-analysis, are needed to identify who may experience adverse effects from self-guided iCBT. IPD meta-analysis allows us to move beyond the ‘grand mean’ to explore change at the individual level as well as to examine study variability (e.g. level of adherence, settings, etc.)

The present IPD meta-analysis examined rates of clinically significant symptom deterioration under self-guided iCBT compared with control conditions in adults with depression. In addition, we examined several socio-demographic and clinical variables as potential moderators of symptom deterioration. In the context of the present paper, the term ‘self-guided iCBT’ is defined as CBT treatment delivered via the Internet without any professional support related to the therapeutic content. The present analysis focuses on the prevalence of deterioration (numbers of persons affected) in contrast to our previous IPD meta-analysis of the present dataset in which we focused on population average outcomes due to self-guided iCBT on depression severity (Karyotaki *et al.* 2017)

## Methods

### Studies selection process

We included IPD from randomised controlled trials comparing self-guided iCBT to a control condition (waiting list, treatment as usual, attention placebo or other non-active controls) for adults (⩾18 years old) with symptoms of depression based either on a diagnostic interview or validated self-report scales. We excluded studies that did not primarily target depression. No limits were applied for language and publication status.

To identify eligible studies, we used an existing database of trials focusing on psychological therapies for adults with depression (Cuijpers *et al.* 2008). This database was developed in 2006 and is updated annually by a systematic literature search in four major bibliographic databases (PubMed, Embase, PsycINFO and Cochrane Library). The database was updated up to 1 January 2016 for the current search. In this search, various index and free terms of psychotherapy were used in combination with index and free terms of depression. Appendix A. presents the full electronic search string for PubMed. Two reviewers (PC and EK) examined 13 384 titles and abstracts independently. All full texts of papers that possibly met inclusion criteria according to one of the two reviewers were retrieved and checked for eligibility. Disagreement on the inclusion was solved through discussion. Additionally, references of recently published meta-analyses on this field were examined to ensure the inclusion of all eligible published trials. Finally, key researchers who are actively involved in this field were contacted to ask whether they were aware of unpublished trials on this topic or trials missed through the searches.

### Data collection process and data items

We contacted the first or the senior author of the RCTs to request access to their datasets. In the case of no response, a reminder email was sent after 2 weeks and after 1 month. If no response was received 1 month after the first email, the study was excluded as unavailable. Authors provided IPD including socio-demographic variables (age, gender, educational level, employment status and relationship status), pre- and post-treatment depression scores, anxiety scores at baseline and the number of iCBT modules completed by each participant. All IPD were merged into one dataset. We also extracted study level variables available from the published reports of the included RCTs (type of control condition, recruitment method and level of support provided).

### Risk of bias assessment

Two independent reviewers (EK and PC) assessed the risk of bias in the included studies according to the Cochrane risk of bias assessment tool (Higgins & Altman, 2008; Higgins & Green, 2011). We examined whether the included studies were at low or high risk of selection, performance, detection, attrition, reporting and other sources of bias. In cases of uncertainty, clarification was sought from the authors of the RCT.

### Measures

The included studies used either the Beck Depression Inventory [BDI-I (Beck *et al.* 1961) or BDI-II (Beck *et al.* 1996)], the Centre for Epidemiological Studies Depression Scale [CES-D (Radloff, 1977)] or the Patient Health Questionnaire [PHQ-9 (Kroenke *et al.* 2001)] as outcome measures of depression severity.

We classified ‘clinically significant deterioration’ according to each participant's reliable change index (RCI) (Jacobson & Truax, 1991), which is the most commonly used method for calculating clinically significant negative effects. This method aims to ensure that each individual's deterioration could not attributable to measurement error (Lambert *et al.* 1993) and thus might warrant clinical intervention in the context of an unsupervised intervention such as iCBT. An RCI of ±1.96 indicates that the difference in scores is likely to be due to a real change in symptoms of an individual (95% confidence level). Participants showing a clinically significant change with an increase in their score (clinically significant negative change of more than −1.96) were classified as clinically significant deteriorated (Jacobson & Truax, 1991). A RCI was calculated separately for each of the studies, using their pre-treatment standard deviation, and the test–retest reliability coefficient of the outcome measure (BDI = 0.93, CES-D = 0.87, PHQ-9 = 0.84).

Finally, in a sensitivity analysis we calculated ‘any deterioration’ as any increase of equal or more than one point on depressive symptom scales (increase ⩾1 BDI or CES-D or PHQ-9 point) from pre- to post-treatments. Any deterioration includes all deterioration rates (clinically significant or not).

### Missing data

Missing outcome data at the post-treatment were multiply imputed. We generated one hundred imputed datasets based on the missing-at-random assumption (‘mi impute mvn’ in Stata version 13.1; StataCorp LP). These datasets, which consisted of the observed and the imputed deterioration rates, were analysed separately using the selected model and the results were combined using Rubin's rules (Rubin, 2004). We ran sensitivity analyses including only observed values to ensure the robustness of the results (‘the complete case analysis’).

## Analysis

### One-stage IPD

We performed a one-stage IPD meta-analysis in which the IPD from all included studies were merged, with participants nested within studies. A one-stage IPD approach is preferred over a two-stage approach because it allows for a more exact likelihood specification (Stewart & Parmar, 1993; Debray *et al.* 2013; Burke *et al.* 2016). We used the statistical software Stata (version 14.2) for conducting all analyses. A multilevel-mixed effects logistic regression was used to analyse the effect of the iCBT intervention on clinically significant deterioration and any deterioration. We used a random intercepts model with a random effect for each trial and fixed effect for condition (intervention *v.* control) using the ‘*melogit*’ command in Stata. The binary variables ‘*clinically significant deterioration*’ and ‘*any deterioration*’ were used as fixed effect.

To explore the variation in outcomes between participants, we examined dichotomous and continuous baseline characteristics of the participants as potential moderators of clinically significant deterioration and of any deterioration. Moderation was tested by adding the interaction between each moderator and deterioration rates to the multilevel mixed-effects logistic regression model. Similarly, we examined whether adherence (defined as number of modules completed divided by the total number of treatment modules) predicted lower deterioration rates within the intervention group.

### Two-stage IPD

In addition to the one-stage IPD, we performed a two-stage IPD to examine the effects of study-level variables. We calculated the odds ratio (OR) of deterioration for each study and we pooled the outcomes using a random effects model, chosen because considerable heterogeneity across studies was expected. The OR shows the probability that an event (deterioration) will occur in the intervention group (self-guided iCBT) compared with the probability that the same event occurring in the control group. An OR of more than 1 indicates increased probability that an event will occur in the intervention group while and an OR <1 shows increased probability that an event will occur in the control group (Deeks *et al.* 2008).

To examine heterogeneity, the *I*^2^-statistic was calculated. This is an indicator of heterogeneity expressed as a percentage: an *I*^2^ value of 0% is interpreted as no heterogeneity, 25% as low, 50% as moderate and 75% as high heterogeneity (Cohen, 1988). We calculated the 95% confidence intervals (CI) around *I*^2^ using the non-central chi-squared-based approach within the heterogeneity module for Stata (Orsini *et al.* 2006; Evangelou *et al.* 2007). Publication bias was examined by visually inspecting funnel plots and by using the Duval and Tweedie's trim and fill method, which yields an estimate of the effect size after adjusting for publication bias (Egger *et al.* 1997; Duval & Tweedie, 2000). We also conducted the Egger's test of the intercept to quantify the bias captured by the funnel plot and test whether it was significant^30^.

Finally, we examined study-level moderators by conducting subgroup analyses using the mixed effects models in which studies were treated as random effects and subgroups were tested as fixed effects.

### Sample representativeness

In our previous analysis (Karyotaki *et al.* 2017), we tested differences between 13 studies that provided IPD and three eligible studies that did not. Results indicated that there are no statistically significant differences in depression severity outcomes between the included and the unavailable trials (Karyotaki *et al.* 2017). This suggests that the present sample of studies is representative. However, we could not test differences between included and unavailable trials in the current analysis since deterioration rates are not reported by the published reports of the unavailable trials.

## Results

### Study selection

**Fig. 1.**
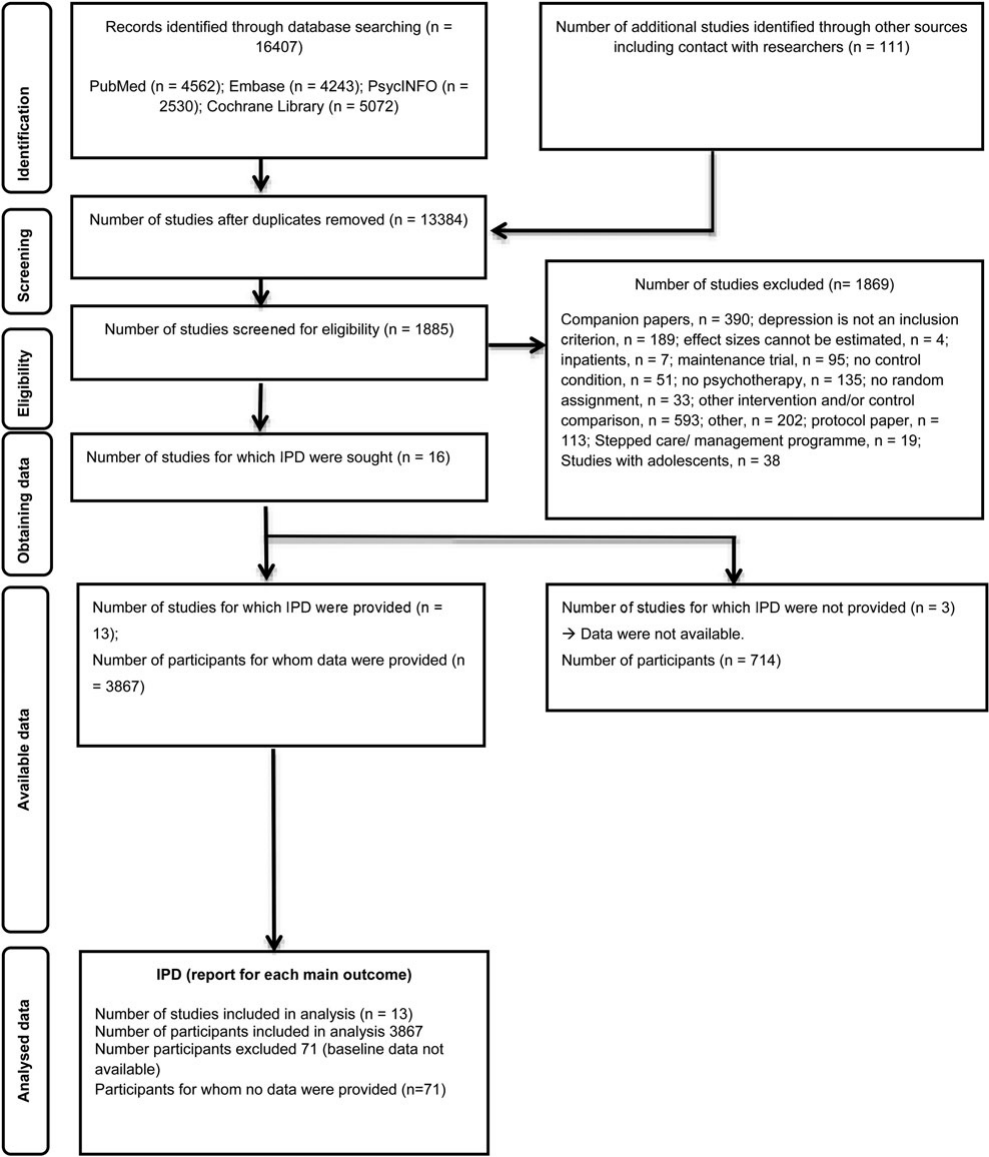
PRISMA IPD diagram of studies selection process.

The systematic literature search resulted in 16 eligible studies for inclusion. The flowchart of the study selection process is presented in Fig. 1. Data were available from thirteen trials including 3876 participants (Christensen *et al.* 2004; de Graaf *et al.* 2009; Berger *et al.* 2011; Farrer *et al.* 2011; Gilbody *et al.* 2015; Karyotaki *et al*. 2017 accepted, Spek *et al.* 2007; Meyer *et al.* 2009; Moritz *et al.* 2012; Phillips *et al.* 2014; Kleiboer *et al.* 2015; Meyer *et al.* 2015; Klein *et al.* 2016; Mira *et al.* 2017). Three eligible datasets were excluded from the present IPD meta-analysis as data were unavailable (Clarke *et al.* 2002; Clarke *et al.* 2005; Clarke *et al.* 2009).

### Studies and participants characteristics

Studies characteristics are presented in Table 1. Across the 13 included RCTs participants were recruited mainly within communities. The studies were conducted in six countries (Australia, Germany, Spain, Switzerland, the Netherlands and the UK). The included RCTs examined the effects of self-guided iCBT over control conditions (attention placebo, no treatment, treatment as usual or waiting list). The interventions consisted of five to 11 online self-guided modules. The majority of the trials provided fully self-guided iCBT while four studies provided technical support related to technical aspects of the web-platform.

**Table 1.** Characteristics of included studies

Study (year)	Inclusion criteria	Outcome measure	Intervention programme	Number of modules	Number of participants – intervention	Technical support	Control condition	Number of participants – controls	Post-treatment assessment	Country
Berger *et al.* (2011)	BDI-II > 13; major depression or dysthymia according to DSM-IV (Mini-DIPS)	BDI-II	Deprexis	11	25	No	WL	26	10 weeks	CH, DE
Christensen *et al.* (2004)	K10 ⩾ 22	CES-D	Moodgym	5	182	No	AP	178	6 weeks	AU
De Graaf *et al.* (2009)	BDI-II ⩾ 16;	BDI-II	Colour Your Life	9	200	No	TAU	100	8 weeks	NL
Farrer *et al.* (2011)	K10 ⩾ 22	CES-D	BluePages; MoodGYM	5	83	Yes	NT	35	6 weeks	AU
Gilbody *et al.* (2015)	PHQ-9 ⩾ 10	PHQ-9	Beating the Blues;	8 5	452	Yes	TAU	239	16 weeks	UK
Kleiboer *et al.* (2015)	39 ⩾ CES-D ⩾ 16; 15 > HADS ⩾ 8	CES-D	Alles Onder Controle	5	107	No	WL	106	6 weeks	NL
Klein *et al.* (2016)^a^	5⩾ PHQ-9 ⩾ 14	PHQ-9	Deprexis	11	192	Yes	TAU	187	12 weeks	DE
Meyer *et al.* (2009)	Completed at least half of the baseline BDI	BDI	Deprexis	11	320	No	WL	76	9 weeks	DE
Meyer *et al.* (2015)	PHQ-9 ⩾ 15	PHQ-9	Deprexis	11	78	No	TAU	85	12 weeks	DE
Mira *et al.* (2017)	BDI-II < 28; experiencing at least one stressful event that produces interference.	BDI-II	Smiling is Fun	8	80	Yes	WL	44	12 weeks	ES
Moritz *et al.* (2012)	Minimal to severe depression: BDI > 0	BDI	Deprexis	11	105	No	WL	105	8 weeks	DE
Phillips *et al.* (2014)	PHQ-9 ⩾ 2 on five of the nine items, including ⩾2 on item 1 or item 2.	PHQ-9	MoodGYM	5	318	No	AP	319	6 weeks	UK
Spek *et al.* (2007)	EDS ⩾ 12; no compliance with the DSM-IV diagnostic criteria of depression (WHO CIDI)	BDI-II	Colour Your Life	10	67	No	WL	58	10 weeks	NL

AP, Attention Placebo; BDI, Beck Depression Inventory; CBT, Cognitive Behavioural Therapy; CES-D, Centre of Epidemiological Studies for Depression Scale; EDS, Edinburgh Depression Scale; HADS, Hospital Anxiety and Depression Scale; IPT, Interpersonal Psychotherapy; K10, Kessler 10 Psychological Distress Scale; Mini DIPS, Mini Diagnostic Interview for Psychiatric Disorders; *n*, number; NT, no treatment; PHQ-9, Patient Health Questionnaire; PST, Problem Solving Therapy; TAU, treatment as usual; WL: waiting list; WHO CIDI, World Health Organization Composite International Diagnostic Interview.

a Klein *et al.* 2016 trial provided therapeutic support to participants with moderate depression (PHQ-9 > 9). Participants with mild depressive symptoms received no support throughout the trial. Klein *et al.* 2016 stratified participants based on depression severity during randomisation. Therefore, we decided to exclude all participants who received therapeutic support (PHQ-9 > 9; *n* = 634) from the present IPD meta-analysis.

Patient characteristics of the intervention and control groups are presented in Table 2. There were no significant between-group differences in gender, age, relationship status, employment, education level, comorbid anxiety or pre-treatment scores on depression measures. Two-thirds of the participants were female (*n* = 2531/3832, 66%), and the mean age was 42 years (s.d. = 11.7). Most of the participants were employed (*n* = 2233/3146, 71%) and had a high school degree or further education (*n* = 2314/2574, 90%). Participants had mean baseline scores of BDI-II = 28.3 (s.d. = 14.4), CES-D = 25.7 (s.d. = 10.8) and PHQ-9 = 14.2 (s.d. = 5.4). A small number of individual cases (*n* = 71) did not start the treatment or did not have baseline data, leaving a total of *n* = 3805. Finally, 997 participants dropped out from the included RCTs and did not provide post-treatment scores of depressive symptoms (26% of the total sample). More specifically, 630/2220 (28.4%) and 347/1585 (22%) participants dropped out of the intervention and controls, respectively. The intervention had significantly higher study dropout rates compared with controls (OR 1.51, 95% CI 1.11–2.08; *p* = 0.01).

**Table 2.** Sociodemographic and clinical characteristics of study participants

Patient characteristics (*n* = 3847)		Intervention group (*n* = 2244)	Control group (*n* = 1603)
Gender female, *n* (%)		1507/2244 (67.4)	1024/1603 (64.2)
Age in years (mean ± s.d.)		41.37 ± 12.27	42.49 ± 11.99
In a relationship, *n* (%)		1239/2124 (58.3)	881/1489 (59.2)
Employed, *n* (%)		1306/1862 (70.1)	927/1255 (73.9)
Education level, *n* (%)	Low	154/1593 (9.7)	106/981 (10.8)
	Middle	869/1593 (54.6)	499/981 (50.9)
	High	570/1593 (35.8)	376/981 (38.3)
Pre-treatment depression scores (mean ± s.d.)	BDI-II	27.60 ± 13.5	29.67 ± 15.9
	CES-D	26.21 ± 10.8	25.25 ± 10.9
	PHQ-9	14.44 ± 5.4	13.8 ± 5.6
Post-treatment depression scores (mean ± s.d.)	BDI-II	20.36 ± 14.4	25.97 ± 16.7
	CES-D	18.6 ± 11.2	22.04 ± 12.2
	PHQ-9	9.19 ± 6.0	9.63 ± 5.9
Comorbid anxiety disorder, *n* (%)		425/972 (43.7)	336/789 (42.6)

### Assessment of risk of bias

The overall risk of bias was low across the included RCTs. Random sequence was adequately generated and allocation was concealed in all included studies. Blinding of participants and personnel is not possible in psychotherapy research due to the nature of the treatment. The included RCTs used self-report outcome measure, thus blinding of outcome assessment is not applicable. As an attempt to minimise attrition bias, we multiply imputed the missing data in the present IPD. Finally, there was no indication of selective reporting and other sources of bias. For a justification of risk of bias assessment for each study, the reader is referred to our previous publication (Karyotaki *et al.* 2017).

### Prevalence of clinically significant deterioration

Overall, 7.2% (276/3805) of participants met criteria for clinically significant deterioration, while 30.1% (1143/3805) showed deterioration of any size. Divided by treatment group, 5.8% of participants who followed self-guided iCBT and 9.1% of participants in control groups showed clinically significant deterioration. Similarly, 26.2% of participants in self-guided iCBT and 35.3% of participants in control groups experienced any deterioration. Complete case analysis showed that 6.1 and 9.1% of participants showed clinically significant deterioration in self-guided iCBT and control groups, respectively. Any deterioration rates were 26.5% for self-guided iCBT and 36.6% for the control groups.

### One-stage IPD: deterioration rates

The outcomes of one-stage IPD meta-analysis on deterioration rates are summarised in Table 3. Self-guided iCBT resulted in significantly lower clinically significant deterioration (OR 0.62; 95% CI 0.46–0.83, *p* < 0.001) compared with controls at post-treatment assessment (6–16 weeks post-randomisation). Sensitivity analysis on any deterioration rates and complete case analyses yielded similar outcomes, suggesting that these findings are robust. No significant associations were found between participant characteristics (age, gender, educational level, relationship status, employment status, comorbid anxiety and baseline severity of depression) or for deterioration rates in both full sample and complete case analyses. Finally, treatment adherence was not significantly associated with clinically significant (*β* = −0.01; s.e. = 0.37, *p* = 0.97) or any deterioration (*β* = −0.10; s.e. = 0.19, *p* = 0.59) within the intervention group.

**Table 3.** Relative odds of deterioration under self-guided iCBT *v.* controls in one-stage IPD analysis

	‘Clinically significant deterioration’						‘Any deterioration’					
	*Full sample*			*Complete cases*^a^			*Full sample*			*Complete cases*^a^		
Variable	*N*_obs_ (*N*_st_)	OR (95% CI)	*p*	*N*_obs_ (*N*_st_)	OR (95% CI)	*p*	*N*_obs_ (*N*_st_)	OR (95% CI)	*p*	*N*_obs_ (*N*_st_)	OR (95% CI)	*p*
Main effects – deterioration												
Treatment group	3795	0.62 (0.46–0.83)	0.001	2818	0.61 (0.46–0.83)	0.001	3795	0.65 (0.55–0.76)	<0.001	2818	0.66 (0.56–0.77)	<0.001
	(13)			(13)			(13)			(13)		
Age												
Treatment group	3786	0.36 (0.13–1.00)	0.05	2809	3.22 (1.10–9.47)	0.04	3786	0.66 (0.36–1.18)	0.16	2809	0.68 (0.38–1.22)	0.20
Age × Treatment group	(13)	1.01(0.99–1.03)	0.27	(13)	1.02 (1.00–1.04)	0.19	(13)	1.00 (0.98–1.02)	0.96	(13)	1.00 (0.98–1.02)	0.93
Gender												
Treatment group	3788	0.61(0.43–.87)	0.008	2811	0.61 (0.42–.88)	0.008	3788	0.61 (0.50–0.74)	<0.001	2811	0.61 (0.50–0.74)	<0.001
Gender × Treatment group	(13)	1.02 (0.58–1.80)	0.96	(13)	1.05 (0.58–1.89)	0.87	(13)	1.20 (0.86–1.67)	0.28	(13)	1.27 (0.89–1.81)	0.18
Educational level												
Treatment group	2538	0.57 (0.23–1.44)	0.23	1973	0.59 (0.23–1.52)	0.28	2538	0.46 (0.26–0.83)	0.01	1973	0.46 (0.25–0.84)	0.01
Educational level × Treatment group	(10)			(10)			(10)			(10)		
Secondary *v.* primary education		1.48 (0.51–4.26)	0.48		0.65 (0.22–1.91)	0.43		1.57 (0.82–2.99)	0.18		1.67 (0.86–3.24)	0.14
Tertiary *v*. primary education		0.99 (0.34–2.91)	0.98		0.90 (0.30–2.71)	0.86		1.27 (0.65–2.48)	0.47		1.25 (0.63–2.47)	0.53
Relationship status												
Treatment group	3568	0.58 (0.37–0.91)	0.02	2630	0.59 (0.38–0.92)	0.02	3568	0.61 (0.48–0.80)	<0.001	2630	0.63 (0.48–0.81)	<0.001
Relationship status × Treatment group	(12)	1.15 (0.64–2.07)	0.63	(12)	1.12 (0.78–1.59)	0.71	(12)	1.02 (0.75–1.40)	0.88	(12)	1.01 (0.71–1.44)	0.96
Employment status												
Treatment group	3067	0.66 (0.40–1.09)	0.11	2194	0.70 (0.41–1.18)	0.18	3067	0.55 (0.40–0.77)	<0.001	2194	0.57 (0.40–0.80)	0.001
Employment status × Treatment group	(10)	0.97 (0.14–6.50)	0.92	(10)	0.92 (0.48–1.76)	0.82	(10)	1.22 (0.83–1.81)	0.32	(10)	1.21 (0.80–1.83)	0.36
Comorbid anxiety												
Treatment group	1728	0.58 (0.37–0.91)	0.02	1447	0.85 (0.37–0.91)	0.02	1728	0.58 (0.43–0.77)	<0.001	1447	0.57 (0.42–.76)	<0.001
Comorbid anxiety × Treatment group	(9)	1.13 (0.57–2.26)	0.72	(9)	1.22 (0.60–2.47)	0.57	(9)	1.04 (0.68–1.60)	0.85	(9)	1.12 (0.71–1.75)	0.64
Baseline severity of depression												
Treatment group	3795	0.61 (0.44–0.85)	0.003	2818	0.61 (0.44–0.85)	0.003	3795	0.65 (0.54–0.77)	<0.001	2818	0.66 (0.56–.79)	<0.001
Baseline severity × Treatment group	(13)	0.99 (0.74–1.33)	0.92	(13)	0.99 (0.72–1.35)	0.93	(13)	1.06 (0.91–1.24)	0.46	(13)	1.11 (0.94–1.29)	0.21

*N*_obs_, Number of observations; *N*_st_, number of studies; OR, odds ratio; s.e., standard error

a This is a sensitivity analysis that was conducted including only participants who completed post-treatment depression questionnaires

### Two-stage IPD deterioration rates

Results of the two-stage IPD meta-analysis replicated the findings of one-stage IPD on clinically significant deterioration (OR 0.62; 95% CI 0.48–0.81, *p* < 0.001). Heterogeneity was zero. These results were replicated in sensitivity analysis on any deterioration rates showed similar outcomes and complete case analyses. There was no indication for publication bias across all the analyses. Finally, the examined study-level variables were not significantly associated with clinically significant and any deterioration. Results of the two-stage IPD are presented in Table 4 in the online supplement.

**Table 4.** Relative odds of deterioration of self-guided iCBT *v.* controls in adults with depressive symptoms, two-stage IPD

Outcomes	‘Reliable deterioration’					‘Any deterioration’				
	*N*	OR (95% CI)^a^	*I*^2^ (%)	95% CI	*p*^b^	*N*	OR (95% CI)^a^	*I*^2^ (%)	95% CI	*p*^b^
Full sample	13	0.62 (0.48–0.81)	0	0–57%	0.000	13	0.63 (0.51–0.77)	41	0–69%	0.000
Subgroups										
Type of control										
TAU *v.*	4	0.76 (0.51–1.13)	15	0–85%	0.16	4	0.68 (0.45–1.03)	63	0–88%	0.62
Other	9	0.51 (0.35–0.75)	0	0–65%		9	0.60 (0.47–0.76)	31	0–68%	
Recruitment										
Community	7	0.47 (0.30–0.74)	0	0–71%	0.29	7	0.56 (0.41–0.75)	36	0–73%	0.39
Community and/or primary care	4	0.76 (0.51–1.13)	15	0–85%		4	0.68 (0.45–1.03)	63	0–88%	
Other	2	0.64 (0.32–1.30)	0	N/A		2	0.75 (0.54–1.03)	0	N/A	
Support										
Pure self-guided iCBT	9	0.61 (0.45–0.83)	0	0–65%	0.82	9	0.59 (0.46–0.77)	48	0–76%	0.34
Technically supported iCBT	4	0.66 (0.39–1.13)	0	0–85%		4	0.72 (0.53–0.96)	16	0–87%	
Complete case	13	0.61 (0.45–0.83)	0	0–57%	0.000	13	0.61 (0.48–0.79)	50	5–74%	0.000
Subgroups										
Type of control										
TAU *v.*	4	0.85 (0.501.44)	36	0–78%	0.06	4	0.69 (0.40–1.19)	74	26–91%	0.56
Other	9	0.43 (0.27–0.70)	0	0–65%		9	0.59 (0.44–0.76)	31	0–68%	
Recruitment										
Community	7	0.40 (0.23–0.70)	0	0–71%	0.16	7	0.54 (0.37–0.79)	47	0–77%	0.69
Community and/or primary care	4	0.85 (0.50–1.44)	36	0–78%		4	0.69 (0.40–1.19)	74	26–91%	
Other	2	0.56 (0.19–1.59)	0	N/A		2	0.67 (0.44–1.01)	0	N/A	
Support										
Pure self-guided iCBT	9	0.60 (0.43–0.85)	0	0–65%	0.85	9	0.57 (0.41–0.78)	53	0–78%	0.34
Technically supported iCBT	4	0.65 (0.34–1.23)	3	0–85%		4	0.73 (0.49–0.80)	40	0–80%	

*I*^2^, heterogeneity index; *N*, number of studies; N/A, not applicable; OR, odds ratio.

a 95% CI 95% confidence intervals; *p*, *p*-value.

b *p* value between groups.

## Discussion

The present IPD meta-analysis aimed to examine the clinically significant symptom deterioration rates of self-guided iCBT compared with controls and to evaluate the moderating effects of deterioration. Of the 3805 participants included in the analysis, 7.2% showed clinically significant deterioration. Self-guided iCBT had low clinically significant deterioration rates (5.8%) and resulted in lower risk of clinically significant deterioration compared with controls at the post-treatment assessment. Similar results were observed in sensitivity analyses. None of the examined participant- and study-level variables moderated clinically significant deterioration.

This is the first meta-analysis to have systematically examined deterioration rates in self-guided iCBT for adults with depressive symptoms. The finding that self-guided iCBT shows lower deterioration rates compared with controls is consistent with previous IPD meta-analysis of 2079 participants on guided internet based interventions (Ebert *et al.* 2016). According to this work, guided web-based interventions resulted in lower risk of clinically significant deterioration compared with controls. Furthermore, guided internet-based interventions showed 3.6% clinically significant deterioration, which is slightly lower but comparable with the present clinically significant deterioration rates of self-guided iCBT (5.8%) (Ebert *et al.* 2016). The deterioration rates found in the present IPD meta-analysis are in line with the deterioration rates observed in face-to-face treatment (5–10%) (Rozental *et al.* 2014) and considerably lower compared with the 12% rate of deterioration reported by a recent meta-analysis examining change under treatment as usual (Kolovos *et al.* 2017).

The present study has several notable strengths; we were able to obtain IPD from 3876 participants from the majority (81%) of the eligible trials. This large number of participants offered us adequate power to detect statistically significant differences between self-guided iCBT and controls on symptom deterioration rates and to examine moderators of clinically significant deterioration. In addition, the current sample of studies appears to be free from critical bias, since the included trials had high methodological quality. Other strengths of the current work are that heterogeneity between studies was small to moderate and there was no indication of publication bias.

Nevertheless, the current findings should be interpreted with caution due to a number of limitations. The present sample of studies is at (relatively low) risk of availability bias since three eligible studies were not available for inclusion. The published reports of these unavailable trials did not include deterioration rates. Thus, traditional meta-analysis of direct comparison between available and unavailable studies was not possible. However, results of a previous analysis showed no difference between the effectiveness of the available and unavailable studies, indicating that the present sample is representative (Karyotaki *et al.* 2017). Moreover, the intervention had significantly higher study dropout rates compared with control conditions. It has been suggested that study dropout could be either related to symptom deterioration or improvement (Rozental *et al.* 2014). On the other hand, higher study adherence rates in controls, such as waiting list, may be associated to the expectation that participants will eventually receive treatment. Unfortunately, it was not possible to examine reasons for study dropout as this information is largely missing from the included studies. In light of this limitation, it should be noted that in the present analysis missing outcome data were multiply imputed based on missing at random assumption. The missing at random assumption underlying multiple imputation is critical as it assumes that those participants for whom post-treatment data were imputed behaved similarly to those with post-treatment data with comparable profiles. Therefore, if individuals dropped out due to deterioration (missing not at random), this would not be captured and a conservative estimate of deterioration is likely. Nevertheless, we performed a complete case analysis to test the robustness of our main full sample analysis and we found no differences between the full sample and complete cases in deterioration rates. Another limitation is that we could not examine several variables that might influence deterioration, such as previous episodes of depression and symptom duration. Individuals who have experienced several episodes of depression in the past and those who have chronic symptoms might be at greater risk of deterioration. Moreover, the majority of the trials included recruited their participants through the community. Thus, the current findings cannot be generalised to patients treated in clinical settings since active care seekers may differ from individuals responding to advertisements.

From a clinical point of view, the present study has important implications. Although many patients prefer psychotherapy to antidepressants (van Schaik *et al.* 2004), pharmacotherapy dominates primary and secondary mental healthcare while psychotherapy is offered to a lesser degree. Self-guided iCBT has the potential to increase the availability and accessibility of psychotherapy and minimise the costs. However, the implementation of self-guided iCBT in clinical practice is hampered by barriers, such as concerns regarding its safety and effectiveness. The present study has demonstrated that self-guided iCBT results in lower risk for symptom deterioration compared with controls (including regular care services) and our previous study showed that is effective in treating depressive symptoms. This suggests that self-guided iCBT could be an alternative to watchful waiting in general practice and it can be disseminated at a large scale in countries where resources of mental healthcare are limited or inaccessible due to other reasons.

Symptom deterioration occurs in self-guided iCBT and study dropout is higher compared with controls; thus, future studies should carefully examine and report deterioration rates and reasons for study dropout. Future research on self-guided iCBT interventions should examine deterioration rates over longer time periods. Moreover, moderators, such as duration of symptoms and previous episodes, should be addressed by future studies to indicate for whom self-guided iCBT might be harmful. More studies from primary care are needed to replicate the current findings with clinical samples.

## Supplementary material

For supplementary material accompanying this paper visit http://dx.doi.org/10.1017/S0033291718000648. click here to view supplementary material
